# Adolescent's perceptions and expectations of parental action on children's smoking and snus use; national cross sectional data from three decades

**DOI:** 10.1186/1471-2458-9-74

**Published:** 2009-03-04

**Authors:** Maria Nilsson, Lars Weinehall, Erik Bergström, Hans Stenlund, Urban Janlert

**Affiliations:** 1 Department of Public Health and Clinical Medicine, Epidemiology and Public Health Sciences, Umeå University, S-901 85 Umeå, Sweden; 2 Research Department, National Institute of Public Health, S-831 40 Östersund, Sweden; 3 Department of Clinical Sciences, Pediatrics, Umeå University, S-901 85 Umeå, Sweden

## Abstract

**Background:**

Parents play a vital role as children develop tobacco behaviours. Many parents feel unsure about their possibility to influence their teenager's lifestyle. Knowledge about young people's acceptance for parental intervention could increase parental involvement. The overall objective of this study was to explore adolescents' perceptions and expectations of parental action regarding children's smoking and snus use, and whether they have changed over time. To see if there were differences whether the adolescent was a tobacco user or not the adolescents' tobacco use was followed; and described to put the findings on their perceptions and expectations of parental action in a context.

**Methods:**

The study used a repeated cross-sectional design, reporting Swedish national data from three decades. Data were collected in 1987, 1994 and 2003 by a questionnaire mailed to homes, in total to 13500 persons. The annual samples, which were random and national representative, consisted of 4500 young people aged 13, 15 and 17 yr, 1500 individuals per age group. The sampling and data collection procedures were done the same way during each survey. Chi2- tests were used to evaluate differences in distributions.

**Results:**

Adolescents in all age groups became more positive toward parental action over time. In 2003, more then 86% of the adolescents, including both smokers and non-smokers, strongly supported parental action on their children's smoking by trying to persuade them not to smoke (94%), by not smoking themselves (87%) and by not allowing their children to smoke at home (86%). Both non-smokers and smokers supported the idea of parental action in a similar way. Reduced pocket money had a weak support (42%), especially from girls. Eighty-nine percent of the adolescents expected their parents to act against smoking and 85% against snus use.

Smoking was stable at 8% in 1987 and 1994 but decreased to 4% in 2003. In 1987 the snus use prevalence was 4% and in 2003 it was 3%. Snus users were mostly boys while few girls had done more than tried snus. More young people in all age groups had never tried smoking compared to the previous studies. In 2003 57% stated that they had never tried smoking.

**Conclusion:**

Adolescent smoking in Sweden has decreased and the proportion who never tried smoking has increased. The results of this study show that a growing majority of adolescents support strong parental intervention to help them refrain from tobacco, but preferably not in a punitive manner. This finding dismisses the notion that adolescents ignore or even disdain parental practices concerning tobacco. Prevention strategies and interventions addressing adolescent tobacco use that involve parents can be improved by using these findings to encourage parents to intervene against their children's tobacco use.

## Background

Adolescence is an important time of life for public health intervention measures, and improved actions to prevent tobacco use are vital during these years. Almost all first use of tobacco occurs before graduation from high school [1]. In a Swedish study, as many as one out of five children reports having ever used tobacco at age 11 [2]. The onset of smoking has long been described as a process that progresses through five stages from preparation, to trying, to irregular use, to regular use, and finally to nicotine dependent smoking [1,3]. Smoking addiction has been defined as smoking at least five cigarettes a day and daily smoking as a prerequisite for being nicotine dependent and experiencing withdrawal symptoms [4]. In the 1970s, Russell assumed that it took years of intermittent smoking to develop dependence with a regular adult type of smoking [5]. Recent studies have challenged these descriptions and suggested that symptoms of nicotine dependence can be found early in the smoking onset process [6-8]. DiFranza et al conclude that the most susceptible youth risk loss of their autonomy over tobacco within a day or two of first inhaling tobacco smoke [9]. Given this risk for rapid development of nicotine dependence and the modest success rates of youth cessation programmes [10], every effort should be made to keep young people from trying tobacco. To be able to model and adjust interventions, it is essential to have knowledge on adolescents tobacco use patterns: when do they start using tobacco?; how does adolescent tobacco use develop and change over time?; who can play a role in preventing adolescent tobacco use?

Young people start using tobacco in a social context where the attitudes towards tobacco, the tobacco behaviour and practices in their homes [11-13], at their schools [14,15], and in their community matter [16]. Consequently, these areas with their different participants are potentially important areas for anti-tobacco socialisation.

A vital role is played by parents as children develop tobacco behaviours. Parents can reinforce positive or negative tobacco behaviours in their child. Their smoking has a direct effect on a child's current smoking and may contribute to initiation, escalation and onset of daily smoking [11,13,17]. Parental anti-smoking socialisation as a way to prevent children from starting to use tobacco has been studied in recent research. One example is the application of home smoking rules which are found to help deter adolescents from smoking [18]. Adolescents with the lowest smoking prevalence are the ones living in homes with smoking bans and in which no member ever smoked. Living in such a home, they are more likely to have quit if they are a smoker [19]. Strict home smoking rules may have an extended effect on young adults as well as on adolescents, provided that they live in parental homes [18]. The parenting style and the quality of the parent-child relationship can also affect adolescent smoking. Parental control [20] as well as parent-child connectedness [21] and parental concern [12], are important in adolescent smoking initiation. High levels of parent-child connectedness has a protective influence on youth smoking, provided the parent being a non-smoker [22,23].

Parents possible impact on adolescent smoking is somewhat inconsistent with the documented view shared by many that adolescence is a time of life with decreased parental influence [24,25]. Anecdotal evidence suggests that many parents perceive others as more influential on their teenager's lifestyle, that they can not do much, and that the child does not want the parents to bother them about their smoking. If these things are believed, it could result in missed opportunities to prevent adolescent tobacco use. If it is possible to document general acceptance among young people for parental intervention, it could increase parental involvement and therefore desirable effects. To gain more knowledge on how parental practices against tobacco are perceived by the young is therefore essential to motivate hesitant parents and legitimize their actions towards tobacco use in their children.

The aim of this study was to assess adolescent's perceptions and expectations of parental action regarding children's smoking and snus use; and if they had changed over time in Sweden (1987, 1994 and 2003). The adolescents tobacco use was followed to study if there were differences whether they were tobacco users or not and to be able to put the findings on their perceptions and expectations of parental action in a context.

## Methods

The study had a repeated cross-sectional design, reporting Swedish data from three decades. In 1987, a national survey was conducted in Sweden by The National Board for Health and Welfare on young people's use of tobacco, their knowledge, attitudes and beliefs [26]. Follow up studies were carried out by The Swedish National Institute of Public Health in 1994 [27] and in 2003 [28].

There were reports before the first study stating that the majority of young people test tobacco during the first half of their teens [29]. To follow and better understand the development of tobacco use, young people aged 13, 15 and 17 were the study target group. The same three age groups were chosen for all three surveys to be able to follow trends over time.

A questionnaire was sent by post to homes each year for a sample of 4500 young people and consisted of 1500 individuals per age group. In total 13500 persons received the questionnaire. The sample was an individual random, national representative sample stratified by age drawn by Statistics Sweden [30] from the total population, ages 13, 15 and 17 years. The sampling procedure was carried out in the same way and the questionnaire was sent out at the same time of the year for each survey. By the use of an individual sampling procedure, a higher statistical power could be achieved as the cluster effects often found in school surveys were avoided. The first questionnaire was followed by two reminder letters to the non-respondents, including a new questionnaire form.

As the target group for the study was not of legal age, a letter was sent to their parents before the questionnaire was sent out to the children providing information on the study. In the letter, the parents' consent was requested using a passive consent procedure informing the parents how to proceed if they did not want their children to participate in the study. The children's right to answer the questionnaire anonymously was emphasized in the letter.

An analysis of the non-respondents was carried out by Statistics Sweden in 2003 using a calibration technique [31]. The full questionnaire was validated prior to data collection by focus group interviews with boys and girls in the same ages as in the study. Some possible validity problems were identified and the questionnaire adjusted accordingly.

In the questionnaire, the adolescents were asked questions about their personal tobacco use, if they thought that parents should try and influence their children's smoking and if their own parents had acted to prevent them from using tobacco. There were five different types of parental influence for the adolescents to respond to by answering 'yes,' 'no' or 'I don't know.' The alternatives were a) trying to persuade their children not to smoke; b) forbidding their children to smoke; c) not allowing their children to smoke at home; d) not smoking themselves; and e) reducing their children's pocket money.

### Definitions

In the study, the definition of "snus" is Swedish moist snuff. When the word tobacco is used, it includes both smoking cigarettes and snus use. The following definitions were used to describe tobacco use: 1) a **smoker **was an occasional or a daily smoker; 2) **an occasional smoker **was smoking every week but not daily; 3) a **daily smoker **was smoking every day; 4) a **non smoker **had never smoked, just tried or had stopped smoking; 5) a **snus user **used snus ranging from <less than a box a week till > than 4 boxes; 6) a **non snus user **had never used snus, had tried or had stopped using it; 7) a **tobacco user **was a smoker and/or a snus user.

### Ethics

Ethics approval was given in 2003 by the Research Ethics Committee at Umeå University, Umeå, Sweden.

### Statistics

Data were analyzed using SPSS (SPSS Inc., Chicago, IL) and Epi Info (CDC, Atlanta, GA). Differences in distributions were evaluated using Chi2-tests. A p-value of less than 0.05 was considered significant.

## Results

The response rate was 67% in 1987, 83% in 1994 and 66% in 2003. The analysis of the non-respondents carried out by Statistics Sweden in 2003 showed that the non-responses could be regarded as random and most likely had not affected the results. The total number and percentages of study participants are shown in Table 1.

**Table 1:** Study participants 1987, 1994 and 2003, reported by age and sex

		boys	%	girls	%	Total	%
13 yr	1987	480	64	451	60	931	62
	1994	617	82	667	89	1284	86
	2003	488	65	538	72	1026	68
							
15 yr	1987	440	59	404	54	844	56
	1994	606	81	661	88	1267	84
	2003	456	61	512	68	968	65
							
17 yr	1987	654	87	604	81	1258	84
	1994	575	77	611	81	1186	79
	2003	454	61	526	70	980	65

Total	1987	1574	70	1459	65	3033	67
	1994	1798	80	1939	86	3737	83
	2003	1398	62	1576	70	2974	66

### Perception of parental action

Over the three study years adolescents in all age groups became more positive toward parental actions to prevent their children from smoking (p < 0.001). The majority of the adolescents answered that parents should try to influence their children's smoking habits, presented in table 2. The alternatives receiving the strongest adolescent support were "persuade", "not allow to smoke at home," and "not smoke themselves." More then 86% approved of all three alternatives. Support for the alternative "forbid their children to smoke" more then doubled, increasing from 26% in 1987 to 59% in 2003 (p < 0.001). Weaker but increasing support was given to the alternative "reduce pocket money." This alternative was not in the questionnaire in 1987, but between 1994 and 2003, support grew from 26% to 42% (p < 0.001).

**Table 2:** Adolescent's perceptions of parental practices on children's smoking.

		13 yr	15 yr	17 yr	Total
By trying to persuade their children not to smoke	1987	92	86	79	86
	1994	94	90	89	91
	2003	96	93	92	94
					
By not smoking themselves	1987	86	86	86	86
	1994	86	86	89	87
	2003	88	86	88	87
					
By not allowing the children to smoke at home	1987	63	64	59	62
	1994	84	82	76	81
	2003	87	87	84	86
					
By forbidding their children to smoke	1987	45	22	12	26
	1994	54	33	23	37
	2003	76	58	44	59
					
By reducing their children's pocket money*	1987	-	-	-	-
	1994	37	23	17	26
	2003	53	39	32	42

Adolescents answering yes in percent, by age, survey year and order of precedence.

All differences over time are statistically significant p < 0.001, with the exception of alternative "By not smoking themselves" with p = 0.038

* The alternative was not in the 1987 questionnaire

The results from 1987 showed that 87% of the non-smokers supported the idea that parents should try to persuade their children not to smoke. This support grew to 93% in 1994 and 95% in 2003 (p < 0.001). Among smokers, this alternative was supported by 67% in 1987 and grew to 81% in 1994 and 84% in 2003 (p < 0.001). For the alternatives "forbid children to smoke" and "reduce children's pocket money," smokers gave much weaker support then non-smokers. The support for parental action against children's smoking was stronger amongst non-smokers (p < 0.001) but both non-smokers and smokers supported the idea in a similar way.

Statistically significant age differences were found for the alternatives "persuade," "forbid," and "reducing pocket money," with the strongest support from 13 year olds (p < 0.001). No age differences were found for "not allowing the children to smoke at home" and "by parents not smoking themselves." Boys were more positive then girls about the alternatives "forbid" and "reducing pocket money" (p < 0.001).

A majority of the adolescents answered that their parents would try to make them stop if they started smoking or using snus. Eighty-nine percent said that their parents would try to make them stop smoking. The non-smokers were more convinced of parental action then the smokers. Among the smokers, 71% reported that their parents would try to persuade them to stop whilst 4% said that their parents would not care about their smoking. Sixty-seven percent of the smokers said that they would be influenced by their parents not wanting them to smoke, and out of those, 30% said that it would influence them a lot.

Considering snus use, 85% expected their parents to act. It was statistically significant that the non snus users expected this to a greater extent then the snus users. But while 71% of the smokers' parents tried to persuade them to quit, only 36% of the snus users had experienced this parental action. Twenty-two percent of the snus users said that their parents did not care about their snus use.

### Smoking and snus use

Smoking prevalence was stable at 8% in 1987 and 1994, but decreased to 4% in 2003 (p < 0.001), the decrease was found in both girls and boys. In 2003, more young people in all age groups had never tried smoking compared to the previous years studied (p < 0.001). In total, 57% stated that they had never tried smoking; 58% of boys and 56% of girls. The smoking prevalences are shown in table 3.

**Table 3:** Adolescent smoking in 1987, 1994 and 2003, in percent by sex and age

		13 yr %		15 yr %		17 yr %		Total % and (N)	
		Boys	Girls	Boys	Girls	Boys	Girls	Boys	Girls
Have never tried	1987	61	68	39	42	30	26	42 (658)	43 (628)
	1994	55	60	37	34	29	23	41 (733)	39 (762)
	2003	77	76	57	56	36	35	58 (797)*	56 (874)*
									
Have tried	1987	35	28	48	35	45	40	43 (672)	36 (513)
	1994	38	33	45	37	44	40	42 (757)	37 (709)
	2003	21	19	35	30	45	36	33 (462)	29 (448)
									
Have smoked but stopped	1987	2	1	3	2	3	3	2 (40)	2 (31)
	1994	3	3	5	6	6	7	5 (80)	5 (101)
	2003	1	1	4	3	5	8	3 (46)	4 (63)
									
Smoke occasionally	1987	2	3	7	13	11	14	7 (108)	10 (144)
	1994	2	3	7	12	10	10	6 (110)	9 (162)
	2003	1	2	2	5	9	9	4 (54)*	5 (84)*
									
Smoke every day	1987	0	0	3	8	11	17	6 (87)	9 (132)
	1994	2	1	6	11	11	20	6 (113)	10 (198)
	2003	0	2	2	6	5	12	2 (31)*	6 (102)*

The results marked * are the statistically significant results (with p < 0.001) stated and discussed in the paper

The smoking pattern changed with age. Almost all 13 year olds were smoke free; 3% reported smoking and more than 75% said that they had never tried smoking. At age 15, 4% of boys and 11% of girls smoked while more then 50% reported never having smoked. At age 17, 14% of boys and 21% of girls smoked and more then 35% had never tried smoking. For all three study years, more girls than boys were daily smokers (p < 0.001).

Smoking prevalence was lower in 2003 compared to the previous years but there was no corresponding change in snus use. A decrease in snus use was noted among boys from 1987 to 1994 (p < 0.01), but there was no difference between 1994 and 2003.

Snus users were mostly boys while few girls had done more than tried snus. Similar to smoking, snus use also increased with age. The snus use prevalence is presented in table 4. Amongst the boys there was a group that both smoked and used snus, 4% in 1994 and 3% in 2003.

**Table 4:** Adolescent snus use in 1987, 1994 and 2003, in percent by sex and age

		13 yr %		15 yr %		17 yr %		Total % and (N)	
		Boys	Girls	Boys	Girls	Boys	Girls	Boys	Girls
Have never tried	1987	74	92	47	79	34	65	50 (780)	77 (1118)
	1994	77	93	49	78	39	66	56 (989)	80 (1519)
	2003	82	92	59	80	48	60	64 (882)	77 (1213)
									
Have tried	1987	21	8	35	19	38	32	32 (504)	21 (305)
	1994	20	7	36	22	34	32	30 (534)	19 (383)
	2003	15	8	29	19	30	35	25 (335)	20 (319)
									
Have used snus but stopped	1987	2	0	5	2	5	1	4 (60)	1 (11)
	1994	1	0	3	0	6	1	3 (55)	0 (6)
	2003	1	0	2	1	3	1	2 (31)	1 (13)
									
Use less then one box/week	1987	2	0	3	0	5	1	4 (58)	0 (5)
	1994	1	0	5	0	3	1	3 (51)*	1 (19)
	2003	1	0	2	0	2	1	1 (19)	1 (10)
									
Use one box or more/week	1987	1	0	10	0	18	1	10 (164)	1 (9)
	1994	1	0	7	0	18	0	8 (151)*	0 (5)
	2003	1	0	8	0	17	3	8 (117)	1 (16)

The results marked * are the statistically significant results (with p < 0.01) stated and discussed in the paper

## Discussion

Studies on adolescents' perceptions of parental actions against their children's tobacco use are rare. In a North American study, adolescents significantly legitimized parent authority to a greater extent for tobacco and alcohol than for conventional and contemporary issues (ie, religion, education, music, clothing) [32]. In the present study, among both smokers and non-smokers, support for parental action against their children's smoking or snus use is substantial and has grown over time. In 2003, 94% supported the concept that parents should try to persuade their children not to smoke, 87% agreed that parents should influence children by not smoking themselves, and 86% thought children should not be allowed to smoke at home. The three alternatives most strongly supported by the young people represent examples of intervention components that have been found fruitful in adolescent smoking prevention research. The alternative receiving the least support was the reduction of pocket money, and this was more pronounced among the smokers. This corresponds with some recent research suggesting caution in using suppressing control and strictness as they do not have any effect in prevention of smoking uptake [13]. Smoking-specific punishments by parents who smoke themselves are even suggested to increase the risk of smoking escalation among adolescents [33]. The introduction of the tobacco legislation and public discussions can be assumed to influence young people so that they have become more positive towards steps taken to prevent or obstruct their use of tobacco.

Darling and Steinberg have demonstrated as part of their theoretical work that adolescents' adjustment varies as a function of their parents' style and suggested that openness to parental influence is important for prediction of outcomes in adolescents [34]. If so, there is a golden opportunity for successful prevention efforts involving parents. A high proportion of the adolescents support parental action to keep them from tobacco use and this can be assumed to make them more open to their parents' influence. Research has shown that parents' anti-smoking socialisation can prevent children from using tobacco [18-23].

A majority of the adolescents surveyed in 2003 said that their parents would try to make them stop if they started using tobacco. The non-tobacco users were more convinced of parental action then were the tobacco users. It was more common that tobacco using adolescents had tobacco using parents, and this may be one of the explanations for this finding. Parents using tobacco are modelling tobacco use and might also find it difficult to practice anti-tobacco socialisation in a consequential manner.

In this study it was much more common that young smokers than young snus users expected their parents to make their child quit. A larger proportion of snus users said that their parents would not care about their child's snus use. Even if there is growing scientific evidence of negative health effects from Swedish snus use, people often tend to compare it with health effects from smoking. In that comparison anything seems less harmful, and this might explain the more passive stand taken by snus users' parents.

With data from three decades, this study demonstrates that smoking has decreased among Swedish adolescents over the studied years. An even larger decrease has been reported in the Swedish adult population [35]. The number of adolescents who had never tried tobacco increased over the studied years. There was also a decrease in adolescent snus use noted. Some have suggested snus use as an explanation for decreased smoking prevalence in Sweden. This study does not support such a conclusion. In a study of Swedish adolescents Galanti et al concluded that snus use in adolescence did not substitute smoking and that the availability of snus might increase nicotine addiction in vulnerable subgroups [36]. In their study as well as in ours the proportion of girls using snus was very low and the results therefore confined to boys snus use. The explanation for the decrease in smoking among adolescents is probably multi-factorial. The first Swedish tobacco legislation with regulations on smoke free environments was introduced in 1993 and made more stringent in 1994. Legislation prohibiting tobacco sales to minors was introduced in 1997 [37]. The legislation, the increased tobacco taxation together with other national tobacco control efforts, and the influence of public opinion probably decreased the social acceptance for smoking and contributed to this reduction in youth smoking. An important societal determinant for smoking is the social acceptance of smoking. A low social acceptance can be assumed to be an important reason for parents wanting to intervene against their children smoking, in particular for parents who smoke themselves.

In 2003, 3% reported being a smoker at age 13. Thus, to prevent tobacco initiation and escalation one will have to pay attention to children's attitudes towards tobacco and early experimentation. This must be done before they enter their teens. The research findings suggesting a risk for rapid development of nicotine dependence early in smoking onset [6-8] stresses the importance of early prevention measures aimed at "not even trying tobacco." It is essential that parents and others who want to intervene are aware of this information.

While smoking decreased during the study, snus use was stable. More boys than girls used snus while more girls were daily smokers. It should be noted that the same gender pattern is seen in adults [35]. Adults, including parents, serve as models for young people in tobacco use [13].

The reports were confined to adolescents reporting about how parents would act if their children used tobacco. Their answers revealed a positive expectation about parental engagement, an expectation that in itself can affect them positively. A limitation of the study was that the sample sizes were too small to include enough girl snus users to be able to draw valid conclusions about girls snus use. A strength of the study was that data were collected over three decades in a manner that allowed comparisons over time. Other strengths were the validation of the questionnaire prior to implementation of the survey and the analysis of non-respondents.

## Conclusion

The results of this study contradict the perception often expressed by parents that their teenage children do not want them to intervene and that they have lost the possibility of influencing their children's lifestyles, including the use of tobacco. This study shows that a majority of adolescents strongly support that their parents should intervene to help them refrain from tobacco, but preferably not in a punitive manner. This support includes both non tobacco and tobacco using adolescents. The finding rejects the notion that adolescents ignore or even despise parental practices concerning tobacco. Prevention strategies and interventions addressing adolescent tobacco use that involve parents can be improved by using these findings to motivate and encourage parents to be active and to intervene against their children's use of tobacco.

## Competing interests

The authors declare that they have no competing interests.

## Authors' contributions

MN was the main author of the manuscript and involved in all aspects of the study. LW, EB, HS and UB provided scientific oversight and feedback throughout the development of the study and the manuscript. All co-authors have seen and approved the final version of the paper and have agreed to its submission for publication.

## Pre-publication history

The pre-publication history for this paper can be accessed here:


